# Supplementary material for: Parp1 deletion rescues cerebellar hypotrophy in xrcc1 mutant zebrafish
Source: bioRxiv. 2024 Nov 25:2024.11.25.625242. Preprint. [Version 1] doi: 10.1101/2024.11.25.625242 (PMC11722395; doi:10.1101/2024.11.25.625242)
Supplement: Supplement 1 [file media-1.pdf]

A

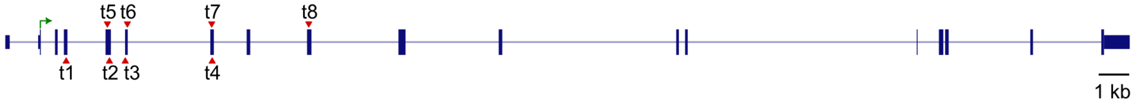

B

gRNA	set	exon	target sequence	genotyping primer F	genotyping primer R	size
t1	1	4	CAGACGTGGAGTGTCCGACAAGG	TCCTCCAGTTGGAAAAGGAAGAG	AACTGACTGAAGACAAAACTCACT	197
t2	1	5	GCTGGTGAAGGCGCAATCACAGG	GCATTGTTTTGTAGGTTCTGCTGG	CCCCTGACCTTGCTGTAGGG	181
t3	1	6	TGTGTGCAGACTATAGCGTACGG	ATGTTTAACCACACCCTTCCAG	TTTAGATGCAAAACACTCACCG	214
t4	1	7	AAAGACGAGTCTCCTTCTGCTGG	GTTCTTCACTTTGAAAGCACCC	GGATTTAGCTGAAGTGTCCCTG	225
t5	2	5	CGTGTGCGTTTCTTCGGGCCCGG	CCGCATGTTTACTCTTCTGCAT	TTCACGCGATCCCACTTCTC	163
t6	2	6	TCACCGGTGGAGTCGAGACTAGG	TATAGCGTACGGCATCTCCTTC	ACACTTTCCGGATGCTCTGTAT	243
t7	2	7	GCTGGGTCAAACGTGCAGCCTGG	ACATCTGTCTGCTCTCTCCCTC	ACTACGGTGCTTTACCCTTCAG	169
t8	2	9	TCGGCAGATGCAGATGAACTGGG	TGAAGCGAAAGTTTGAGTTCAG	TTCATTTGTACCTCTGCTGCTG	152

C

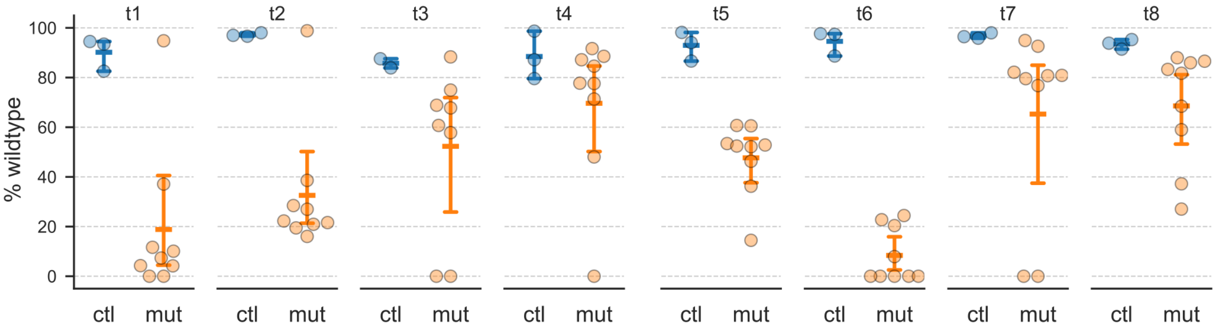

Supplemental Figure 1. Active guide RNAs targeting *xrcc1*

A. Location of guide RNAs tested for efficacy against *xrcc1* exons. The first set of gRNAs (t1-4) were co-injected in the first experiment, and the second set (gRNAs t5-8) co-injected for the replication experiment. B. gRNA target sequence, genotyping primers and PCR product size (base pairs). C. Proportion of PCR product present in the wildtype sized ABI peak in individual embryos for controls that were injected with Cas9 protein and tracR RNA only (blue) or Cas9 protein, tracR RNA and targeting gRNA (orange).

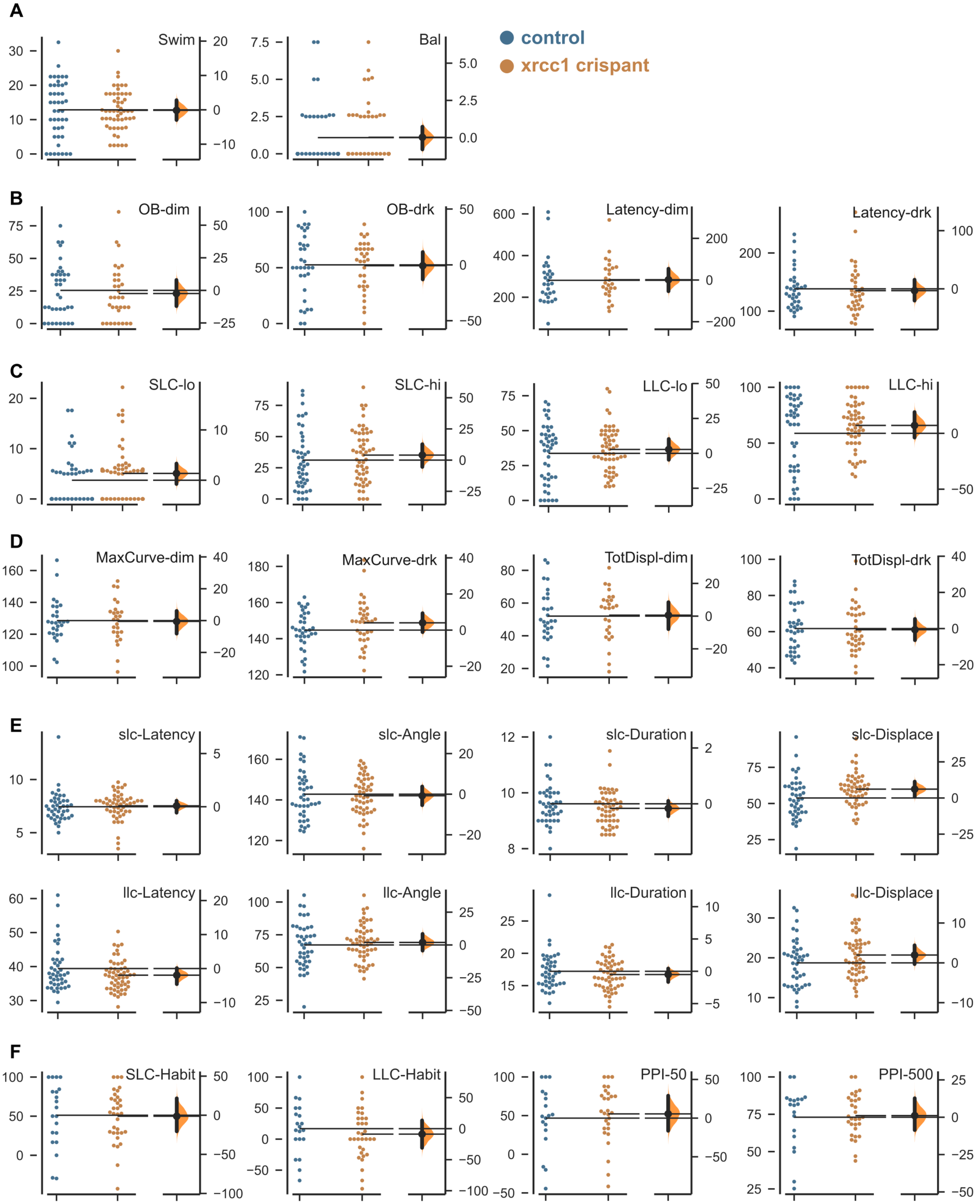
A
Swim
Bal
control
xrcc1 crispant
B
OB-dim
OB-drk
Latency-dim
Latency-drk
C
SLC-lo
SLC-hi
LLC-lo
LLC-hi
D
MaxCurve-dim
MaxCurve-drk
TotDispl-dim
TotDispl-drk
E
slc-Latency
slc-Angle
slc-Duration
slc-Displace
llc-Latency
llc-Angle
llc-Duration
llc-Displace
F
SLC-Habit
LLC-Habit
PPI-50
PPI-500

Supplemental Figure 2. Sensory and motor behavior in *xrcc1* crispants

Estimator plots showing measurements of sensory, motor and central integration in controls (blue) and *xrcc1* crispants (orange). Tests measure spontaneous swimming and balance (A), visual sensory responsiveness (B), auditory sensory responsiveness (C), movement kinematics after visual cue (D), movement kinematics after auditory cue (E) and sensorimotor processing (F). Estimator plots show mean measurements for each larva tested on the left side, and mean difference between controls and crispants on right. The slc-Displace difference was nominally significantly different (p=0.023), but not after adjustment for multiple comparisons. See supplemental table 1 for explanation of measurements. N=18 to 54 larvae per group.

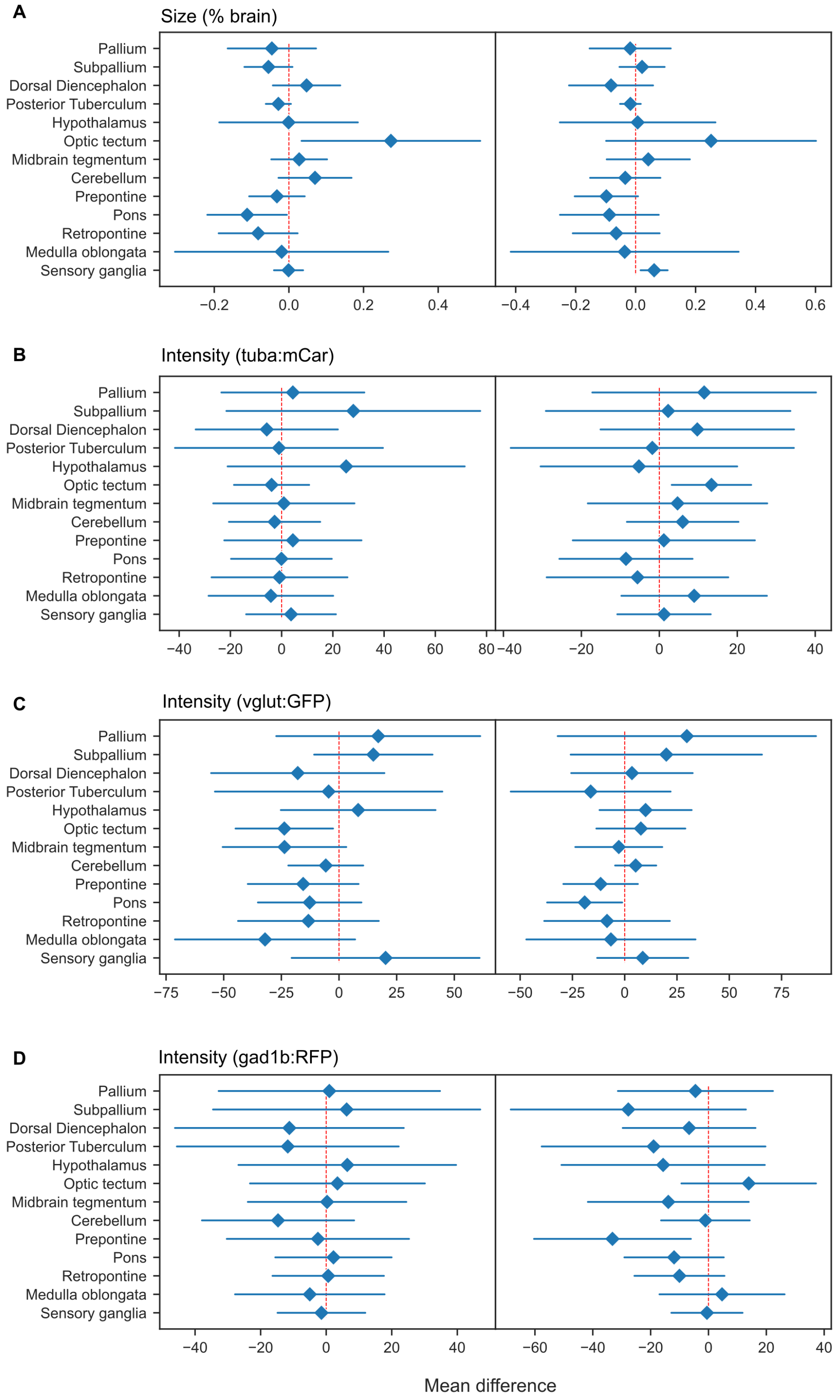

A
Size (% brain)
B
Intensity (tuba:mCar)
C
Intensity (vglut:GFP)
D
Intensity (gad1b:RFP)
Pallium
Subpallium
Dorsal Diencephalon
Posterior Tuberculum
Hypothalamus
Optic tectum
Midbrain tegmentum
Cerebellum
Prepontine
Pons
Retropontine
Medulla oblongata
Sensory ganglia
Mean difference

Supplemental Figure 3. Brain morphometric measurements in *xrcc1* crispants

Mean difference and confidence interval for size (A) and fluorescence intensity (B-D) in brain divisions in *xrcc1* crispants. Division size was normalized as the percentage of total brain volume. Fluorescence intensity is in arbitrary units. Left panels show data for gRNA-t1/2 crispants, right panels for gRNA-t5/6. Mean difference values greater than zero indicate a larger size or greater intensity in controls.

A

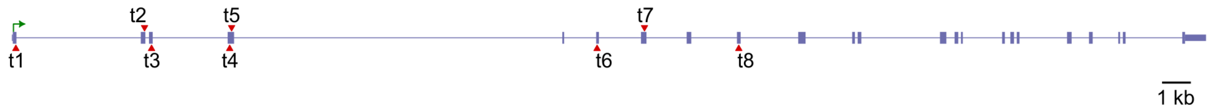

B

gRNA	set	exon	target sequence	genotyping primer F	genotyping primer R	size
t1	1	1	TGCTAAGGACTCGCTGAGGA	CAGGACGACAAGCTGTACAAAG	CCAAAAAGTTGCACACAACAAT	144
t2	2	2	GTGAAGACAGCCATTGAGAG	ATTTACTGACCTCCGCTGGG	AAGACTAACTGCTCACCAGCC	150
t3	2	3	TTTCTGATCGCAGCCCTTGC	ATCCTTGTTGCTTTACAGGGAA	AGCATTGGGCCTAAGTAATTCA	207
t4	1	4	CCGGCTGCTTTGTGAGTCGT	TTTTTGTTGTTGTTCCCTGATG	CTCGTAGAACTGCGAATCCTTT	193
t5	2	4	AGACCAAGCTGAGGTTTCTC	TTTTTGTTGTTGTTCCCTGATG	CTCGTAGAACTGCGAATCCTTT	193
t6	1	6	CCAGCTGATCTGGGGAATTA	CCTTTCTTCCTCATTCTGATGC	GCATTCTTACATTTGACTCCCC	273
t7	2	7	AAGCCTTGTGAGACTTGCAA	TGTGGATCGACTATCAGACTGC	CCGCTTGAAGGAGAGAAAATAG	267
t8	1	9	TACTGAGCTTTCCCACAGCC	TACTTGGCTAAACTGACTGCGA	GATTTTTCCTCCCAGATCTTCC	284

C

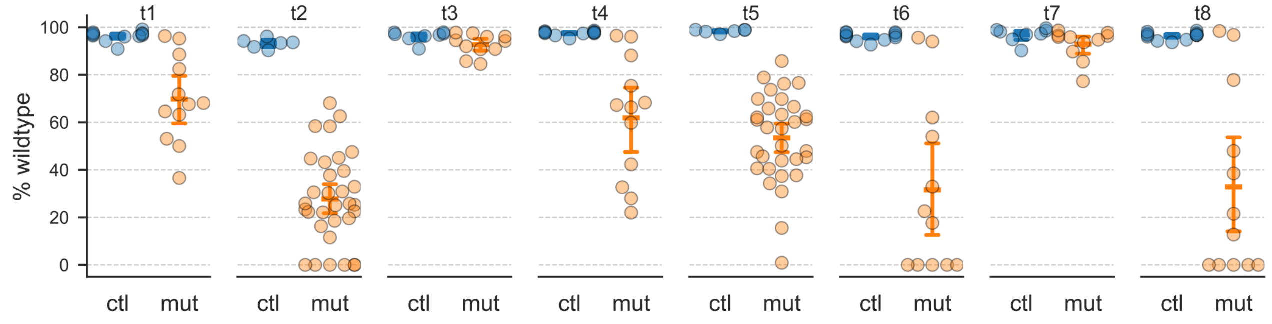

Supplemental Figure 4. Active guide RNAs targeting *parp1*

A. Location of guide RNAs against *parp1* exons tested for cutting efficiency. B. Target sequence and PCR primers for CRISPRstat genotyping for *parp1* guide RNAs. C. Cutting efficiency for *parp1* guide RNAs, shown as the percentage of the normal wildtype peak remaining after injection of each guide RNA during CRISPR experiments.

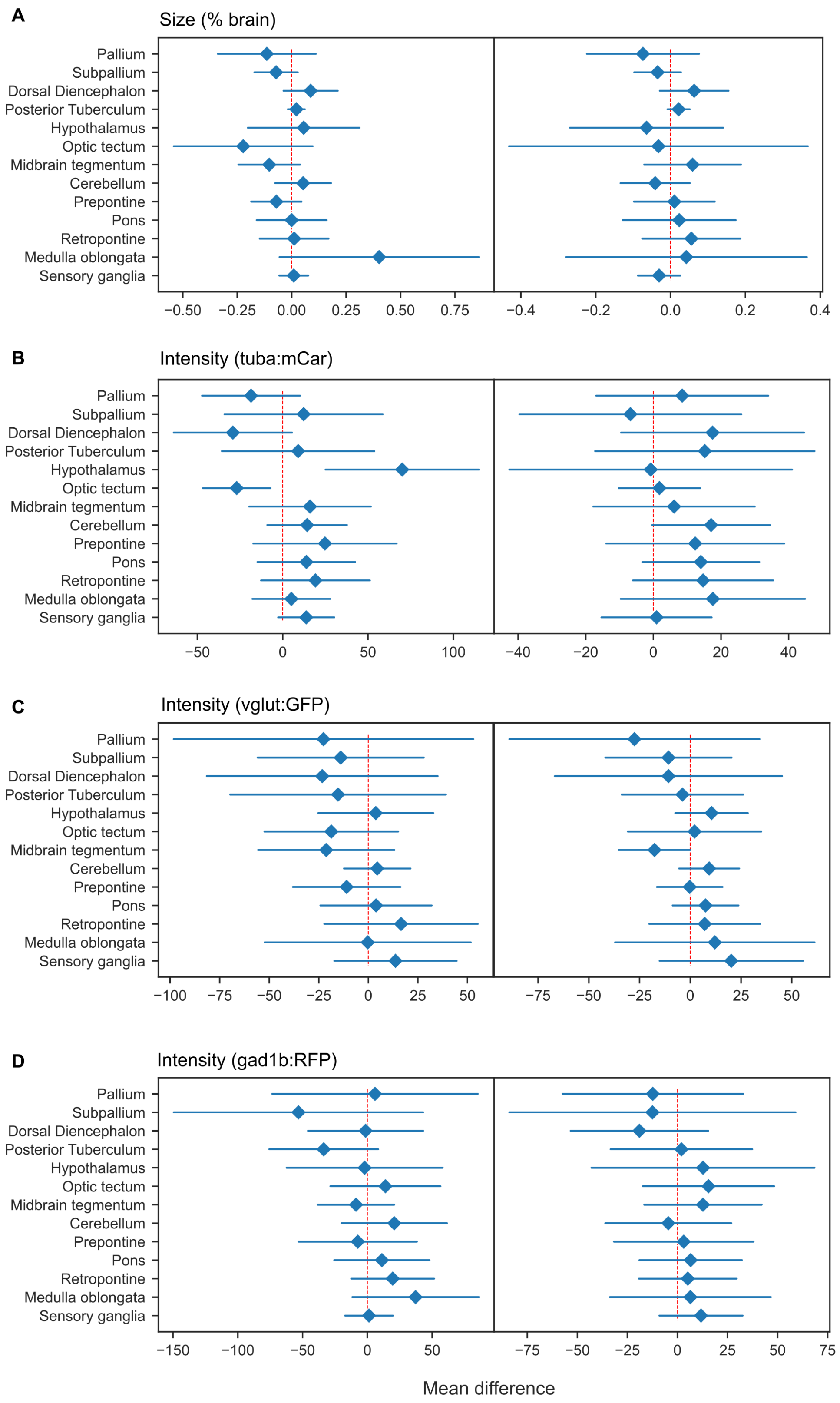
A
Size (% brain)
B
Intensity (tuba:mCar)
C
Intensity (vglut:GFP)
D
Intensity (gad1b:RFP)
Pallium
Subpallium
Dorsal Diencephalon
Posterior Tuberculum
Hypothalamus
Optic tectum
Midbrain tegmentum
Cerebellum
Prepontine
Pons
Retropontine
Medulla oblongata
Sensory ganglia
Mean difference

Supplemental Figure 5. Brain morphometric measurements in *parp1* crispants.

Mean difference and confidence interval for size (A) and fluorescence intensity (B-D) in brain divisions in 6 dpf *parp1* crispants. Left panels: gRNA-t2/5. Right panels: gRNA-t6/8.

Label	Meaning	Measurement
Swim	Spontaneous swimming activity	Percent larvae active in 30 ms window before presentation of auditory cue
Bal	Balance	Percent larvae failing to present with dorsal side up
OB-dim	Visual responsiveness (dim flash)	Percent larvae responding to small light decrement with O-bend
OB-drk	Visual responsiveness (dark flash)	Percent larvae responding to light extinction with O-bend
Latency-dim	Visual responsiveness (dim flash)	Response latency (ms) for larvae with O-bend response to small light decrement
Latency-drk	Visual responsiveness (dark flash)	Response latency (ms) for larvae with O-bend response to light extinction
SLC-lo	Auditory responsiveness (weak auditory cue)	Percent larvae responding to weak auditory cue with short latency C-start
SLC-hi	Auditory responsiveness (intense auditory cue)	Percent larvae responding to intense auditory cue with short latency C-start
LLC-lo	Auditory responsiveness (weak auditory cue)	Percent larvae responding to weak auditory cue with long latency C-start
LLC-hi	Auditory responsiveness (intense auditory cue)	Percent larvae responding to intense auditory cue with long latency C-start
MaxCurve-dim	Movement kinematics (dim flash)	Mean maximal curvature (degrees) during O-bend responses to small light decrement
MaxCurve-drk	Movement kinematics (dark flash)	Mean maximal curvature (degrees) during O-bend responses to light extinction
TotDispl-dim	Movement kinematics (dim flash)	Mean net displacement (pixels) during O-bend responses to small light decrement
TotDispl-drk	Movement kinematics (dark flash)	Mean net displacement (pixels) during O-bend responses to light extinction
slc-Latency	Movement kinematics (short latency c-starts)	Mean latency (ms) to initiation of short latency C-start in response to auditory cue
slc-Angle	Movement kinematics (short latency c-starts)	Mean maximal change in head orientation (degrees) during initial bend of short latency C-start to auditory cue
slc-Duration	Movement kinematics (short latency c-starts)	Mean time (ms) until maximal change in head orientation during initial bend of short latency C-start to auditory cue
slc-Displace	Movement kinematics (short latency c-starts)	Mean net displacement (pixels) during short latency C-start to auditory cue
llc-Latency	Movement kinematics (long latency c-starts)	Mean latency (ms) to initiation of long latency C-start in response to auditory cue
llc-Angle	Movement kinematics (long latency c-starts)	Mean maximal change in head orientation (degrees) during initial bend of long latency C-start to auditory cue
llc-Duration	Movement kinematics (long latency c-starts)	Mean time (ms) until maximal change in head orientation during initial bend of long latency C-start to auditory cue
llc-Displace	Movement kinematics (long latency c-starts)	Mean net displacement (pixels) during long latency C-start to auditory cue
SLC-Habit	Sensorimotor filtering	Percent habituation of short latency C-start responses across trials conducted with intense auditory cue
LLC-Habit	Sensorimotor filtering	Percent habituation of long latency C-start responses across trials

		conducted with intense auditory cue
PPI-50	Sensorimotor filtering	Percent prepulse inhibition of short latency C-starts when intense auditory cue preceded at 50 ms by weak auditory prepulse
PPI-500	Sensorimotor filtering	Percent prepulse inhibition of short latency C-starts when intense auditory cue preceded at 500 ms by weak auditory prepulse

Supplemental Table 1

Definitions of abbreviations for measurements of sensory and motor measurements in supplemental figure 2.
